# Somatostatin analogues for refractory diarrhoea in familial amyloid polyneuropathy

**DOI:** 10.1371/journal.pone.0201869

**Published:** 2018-08-30

**Authors:** Michael Collins, Anna Pellat, Guillemette Antoni, Hélène Agostini, Céline Labeyrie, David Adams, Franck Carbonnel

**Affiliations:** 1 AP-HP, Hôpital Bicêtre, Department of Gastroenterology, Le Kremlin Bicêtre, France; 2 INSERM SC10-US19, Villejuif, France; 3 AP-HP, Hôpital Bicêtre, Unité de recherche clinique Paris-Sud, Le Kremlin-Bicêtre, France; 4 French National Reference Centre for FAP (NNERF), Le Kremlin Bicêtre, France; 5 AP-HP, Hôpital Bicêtre, Department of Neurology, Le Kremlin Bicêtre, France; 6 INSERM UMR 1195; Paris Sud University, Le Kremlin Bicêtre, France; Public Library of Science, UNITED KINGDOM

## Abstract

**Introduction:**

Familial amyloid polyneuropathy (FAP) is a genetic disease leading to the production of a variant transthyretin (TTR) or a beta variant β2-microglobulin. FAP may be associated with refractory diarrhoea. In this study, we assessed the efficacy and tolerance of somatostatin analogues in refractory diarrhoea associated with FAP.

**Methods:**

FAP patients from the French national referral center who received somatostatin analogues for a refractory diarrhoea were retrospectively studied. We assessed remission of diarrhoea, as defined by a stool consistence of five or less on the Bristol stool scale, assessed after three to six months of follow-up. Stool frequency and continence before and after three to six months of treatment were also compared by the means of Wilcoxon and McNemar's exact tests, respectively.

**Results:**

Fourteen patients treated with somatostatin analogues were evaluable. After three to six months of follow-up, 9/14 patients (64% 95%CI = [35%; 87%]) had remission of diarrhoea. This was significantly higher than a theoretical remission rate of 20% (p = 0.0004). There was a significant decrease of daily bowel movement from 6 to 2.5 per day (p = 0.002). Twelve/14 (85%) patients had incontinence at baseline *vs* 8/14 (57%) after three to six months of follow-up (p = 0.134). Three out of 14 patients (21%) had a severe adverse event; two patients had hypoglycaemia, and one had endocarditis due to an injection-site bacterial infection.

**Conclusion:**

This study suggests that somatostatin analogues may benefit to patients with FAP and refractory diarrhoea. Approximately 20% of patients had severe adverse events, including hypoglycaemia.

## Introduction

Familial amyloid polyneuropathy (FAP) is a genetic systemic disease usually due to mutations of the transthyretin (TTR) gene (TTR-FAP) and sometimes to beta variant β2-microglobulin [1]. These mutations lead the liver to produce an unstable protein which accumulates within the nerves, heart, gut and kidney [2–4]. Liver transplantation can stop the progression of the disease but does not deplete tissue TTR [5–7]. Recently, transthyretin tetramer stabilizers have shown their ability to slow progression of the disease [8]. Another class of drugs TTR gene silencing, short interfering RNA (siRNA) or antisense oligonucleotide (ASO) acts by major TTR knockdown, and has shown promising results in a phase II trial; phase 3 clinical trials are ongoing [9,10].

The first manifestations of FAP are generally those due to peripheral sensitive neuropathy or gastrointestinal (GI) symptoms [11]. Gastrointestinal symptoms are constipation, diarrhoea, nausea, vomiting, abdominal pain, dysphagia and faecal incontinence. These symptoms severely deteriorate patients’ quality of life and may lead to malnutrition. The incidence of GI symptoms increases with time. One fourth of patients with FAP have chronic diarrhoea after five years of follow-up [12]. Current anti-diarrhoeal therapy includes loperamide, low fiber intake, and opioids [11,13]. However, many patients fail to improve with these treatments.

The mechanism of diarrhoea in FAP is poorly understood. Intestinal dysmotility due to amyloid enteric neuropathy may contribute to diarrhoea [14], as observed in diabetes-related neuropathy [15]. Loss of neuroendocrine cells, including somatostatin-secreting cells within the intestinal mucosa has also been reported as a putative mechanism of diarrhoea in FAP patients [16–19]. Therefore, somatostatin analogues may be proposed, to treat patients with FAP and refractory diarrhoea. Somatostatin analogues have shown some efficacy in patients with intestinal dysmotility, such as systemic sclerosis [20,21] and in refractory diarrhoea due to other causes [22], such as HIV infection [23,24], and chemoradiotherapy [25]. Somatostatin analogues are widely used in other clinical setting, such as portal hypertension, neuroendocrine tumors and pancreatic fistulas; their main side effects are glycaemic disorders and gallstone disease. The aim of this study was to assess the efficacy and safety of somatostatin analogues in the treatment of refractory diarrhoea in FAP.

## Patients and methods

We conducted a retrospective, observational study over a 2 year-period (2014–2016), in the national referral centre for FAP patients in France. Many patients are addressed from secondary or tertiary care centres, throughout Metropolitan France and French overseas territories.

### Patients

Patients were followed in the neurology department, and were addressed to the gastroenterology department if they had GI symptoms. They were routinely investigated for differential diagnosis, and were subsequently followed-up for evolution, in both the neurology and gastroenterology wards.

### Data collection

We performed a systematic chart review of patients with FAP who were seen at least once in the gastroenterology department of the Bicêtre hospital. Then, we selected adult patients with chronic diarrhoea, as defined by a Bristol stool scale (BSS) of 6 or 7, which is routinely used and reported in gastroenterology consultations within our department. Diarrhoea was defined as refractory when it persisted despite the prescription of conventional anti-diarrhoeal drugs, including loperamide. We selected all patients with refractory diarrhoea who had received somatostatin analogues as a treatment for diarrhoea. We excluded patients who were prescribed somatostatin analogues to treat neuroendocrine tumors.

### Efficiency evaluation

We defined remission of diarrhoea as a BSS value of less than six, as recommended by FDA guidelines (http://www.fda.gov/…/Guidances/UCM205269). We also assessed the variation in stool frequency, body weight and faecal incontinence. Faecal incontinence was defined as, at least one uncontrolled defecation during the week before evaluation. All the variables were assessed at an early (less than 1 month) and a more distant time point (3 to 6 months).

### Safety evaluation

Adverse events were assessed from medical charts of both gastroenterology and neurology departments. We extended safety evaluation to the time of submission of the study. In each case, we evaluated if the adverse event was attributable to somatostatin analogues or to another cause. Severe adverse events were defined as any adverse event that resulted in hospitalization or increased duration of the hospital stay, was fatal or life-threatening, or led to significant disability. We focused on expected toxicity of somatostatin analogues, knowingly glycaemic disorders and gallstone-related disease.

### Statistical analysis

Descriptive statistics were used to analyse baseline characteristics. Continuous variables were described by median and extreme values. Categorical variables were described by frequencies and percentages. The proportion of patients with remission of diarrhoea, as defined above, was assessed after 3 to 6 months of follow-up and the exact 95% Confidence Interval was calculated, based on the cumulative probabilities of the binomial distribution. In the absence of a control group, we compared the remission rate to a theoretical rate of 20% with a binomial unilateral exact test, assuming that a spontaneous improvement would not occur in more than 20% of the patients. This kind of analysis is used in single arm phase II trials [26]. We compared the stool frequency before and after treatment, by the Wilcoxon signed rank test, using each patient as his own control. We used the exact Mc Nemar's test to compare the proportion of continent patients before and after treatment. The tests were bilateral and we considered a p value of 0.05 or less to be statistically significant. All the statistical analyses were done on R3.2.1 version.

### Ethics

This was a non-interventional, retrospective study. Personal data were collected and managed according to national guidelines and laws.

The protocol was approved by local ethic committee, “Comité de Protection des Personnes Ile-de-France VII Bicêtre” (n° 16–044). According to French law, written consent wasn’t mandatory for this monocentric retrospective non interventional study. Therefore, written non-opposition form and information sheet was sent to patients via postal mail to give them opportunity to oppose the use of their data.

## Results

### Patient selection

**Fig 1:**
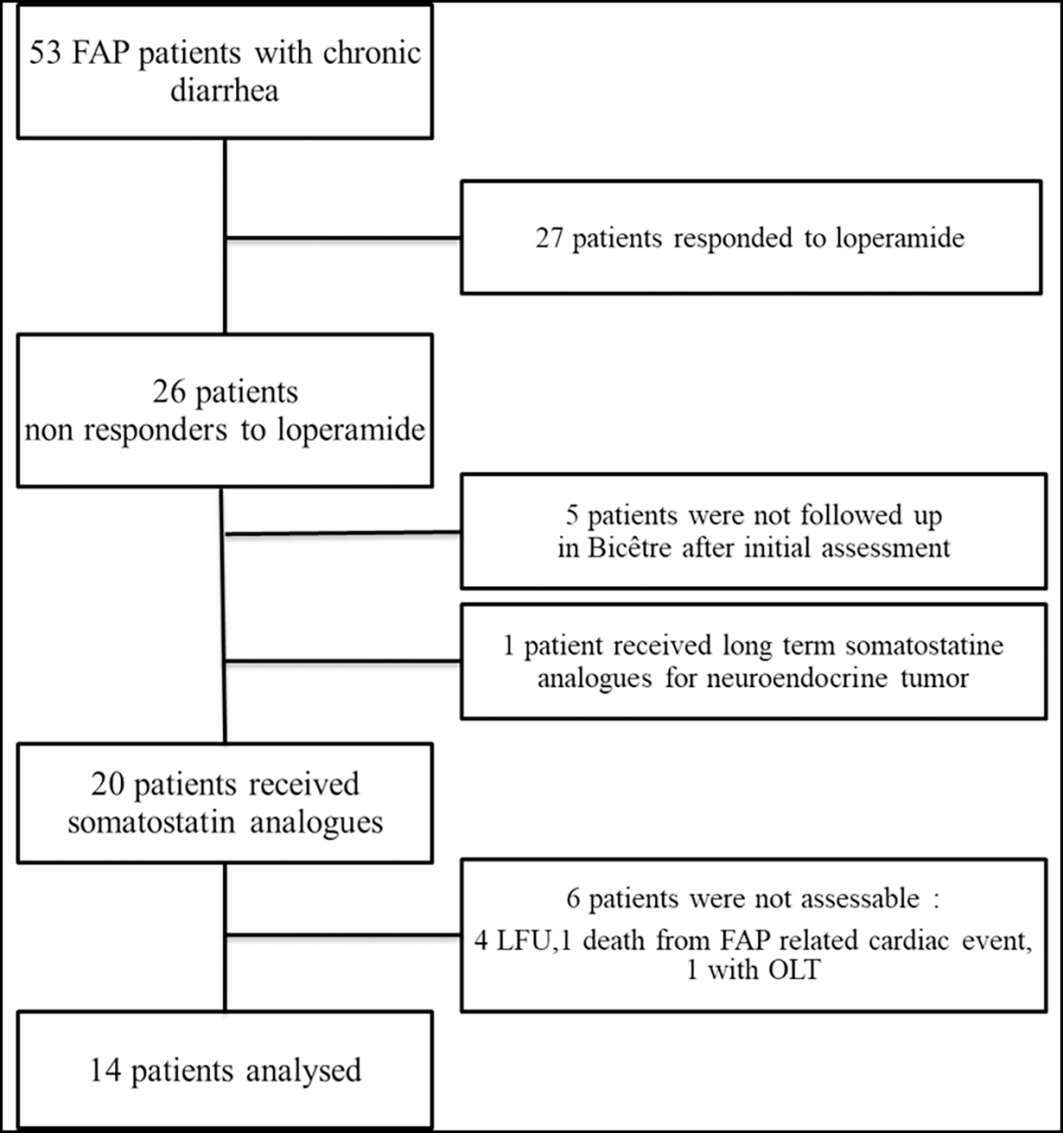
Flow chart of patients with familial amyloid neuropathy and chronic diarrhoea. LFU: lost to follow up NET: Neuro endocrine tumor. OLT: Orthotopic Liver Transplantation.

Between 2014 and 2016, 74 FAP patients were evaluated for gastrointestinal symptoms. Among them, 53/74 (72%) had diarrhoea, 31/74 (41%) had faecal incontinence, 18/74 (24%) had nausea or vomiting, 13/74 (18%) had constipation, 3/74 (4%) had dysphagia, and 13/74 (18%) had other symptoms. All patients with incontinence also had diarrhoea. Therefore, 31/53 (57%) patients had incontinence among those with diarrhoea. Patients’ flow chart is presented in Fig 1.

Among 53 patients with FAP and diarrhoea, 26/53 (49%) patients were refractory to conventional anti-diarrhoeal drugs. Somatostatin analogues were prescribed as compassionate treatment in all but five of these patients, who were not followed-up in Bicêtre after initial assessment. One patient was receiving somatostatin analogues long before diarrhoea, because of a neuroendocrine tumour and was therefore excluded. Among the twenty remaining patients, six patients were not evaluable for the primary outcome. Among these six patients, one patient had an orthotopic liver transplantation (OLT) during the month following treatment initiation, one patient died from cardiac complication of FAP unrelated to somatostatin analogue and four patients were lost to follow up. Therefore, 14 patients with FAP and refractory diarrhoea were treated with somatostatin analogues and had an adequate follow-up. The demographic characteristics of these patients are shown in Table 1. Thirteen had a mutant TTR, one a beta variant β2-microglobulin.

**Table 1:** Demographic characteristics of patients treated with somatostatin analogues for familial amyloid neuropathy and refractory diarrhoea.

Total population	N = 14
**Age (years)**	52.1 [47;120]
**Female gender**	8 (57)
**Transthyretin mutation**	13(92)
- **Val30Met**	10 (71)
- **Non Val30**	4 (29))
**Weight (kg)**	
- premorbid*	68 [47;90]
**- at baseline before somatostatin analogue**	54.5 [35;76,5]
**Time since FAP diagnosis (years)**	6 [2; 21]
**Time with diarrhea (years)**	4 [2; 14]
**Anti amyloid treatment**	
**- Tafamidis**	3 (21)
**- RNAI or antisens oligonucleotids**	4 (28)
**- none**	7 (48)
**Liver transplantation recipients**	4 (29)
**Other FAP-related Symptoms**	
**- Peripheral neuropathy**	14 (100)
**- Cardiac**	12 (86)
**- Glaucoma**	3 (21)
**- Renal failure or proteinuria**	1 (7)
**- Urinary incontinence**	5 (36)
- Erectile dysfunction**	3 (50)
**Latest PND score**	
**- I or II**	9 (65)
**- III or IV**	5 (35)

Quantitative variables appear as Median [extremes] and qualitative variables appear as frequency (%).

PND stands for PolyNeuropathy Disability score, ranging from 0 (no disability) to IV (severe disability).

*1 missing data

** calculated only on the male gender subgroup

### Treatment received

Three patients out of 14 (21%) received slow-release octreotide 30 mg from the start. Treatment was started in 11/14 (79%) patients by subcutaneous (SC) octreotide, at a dose of either 100 μg t.i.d. (8/14; 57%), or 50 μg b.i.d (1/14; 7%) or 50 μg t.i.d (2/14; 14%), for one to three days. Subsequently, one patient out of 11 remained on Octreotide SC, and ten patients had slow release somatostatin analogues once every four weeks. Subcutaneous Lanreotide, 120 mg was administered in 2/10 patients, and intramuscular slow release Octreotide was administered at a dose of 30 mg in 7/10 patients, or 10 mg in 1/10 patient. Concomitant drugs prescribed were loperamide, in 12/14 patients (86%), siRNA or antisense oligonucleotide in 5/14 patients (36%).

### Efficacy

Fourteen patients had complete dataset at baseline and at 3–6 months. A summary of outcomes is available in Table 2.

**Table 2:** Outcomes with somatostatin analogues, at one month and at 3–6 months of treatment.

	Baseline (N = 14)	At 1 month (N = 10)	At 3–6 months (N = 14)
**Remission of diarrhea (n (%))**	-	6/10 (60)	9/14 (64)
**Bristol Stool Scale (Median [range])**	7 [6 ; 7]	5 [3 ; 7]	5 [2 ; 7]
**Number of stools per day (Median[range])**	6 [2 ; 13]	2 [1;6]	2 [0.2;4]
**Patients with fecal incontinence n (%)**	12/14 (86%)	6/9 (67%)	8/14 (57%)
**Body weight in kilogram (Median[range])**	54.5 [35; 76,5]	57.8 [45;76]	55 [35; 76]

Remission of diarrhea at 3–6 months is statisticaly higher than a theoretical proportion of 20% using a binomial exact unilateral test p <0.001. Quantitative variables appear as Median [extremes] and qualitative variables appear as frequency (%).

#### Remission of diarrhoea

**Fig 2:**
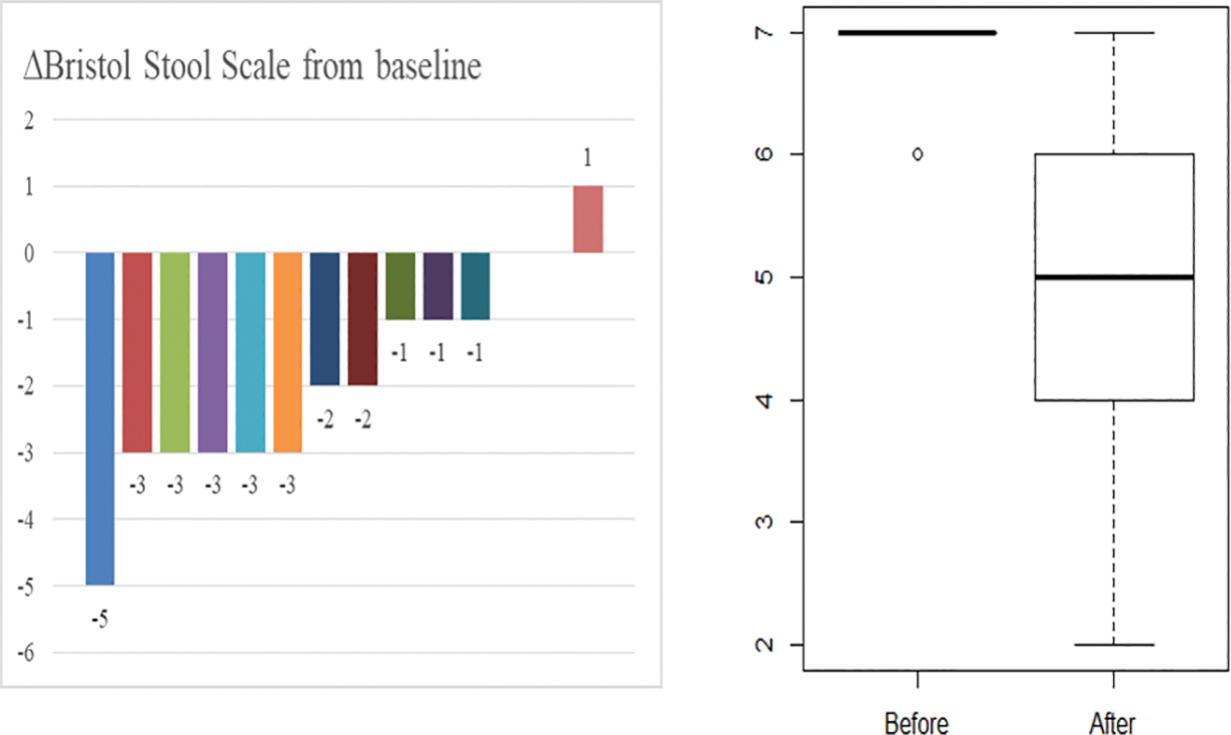
Bristol Stool Scale (BSS) at 3 to 6 months of follow up compared to baseline. BSS ranging from 1 (hard and round shaped stools) to 7 (watery stools). (A)Numerical variation of Bristol stool scale for each individual patient. (B) Boxplot Bristol Stool Scale at baseline (Before) and at 3 to 6 months of treatment (After).

Nine out of 14 patients (64%; 95%CI = [35%; 87%]) had a remission of diarrhoea (BSS lower than 6) after 3 to 6 months of somatostatin analogues. The observed remission rate of 64% (IC95% [0.31; 0.86]) after 3 to 6 months of somatostatin analogue is significantly higher than a theoretical proportion of 20% (binomial exact unilateral p<0.001). Five out of 14 patients (36% IC 95%: [13%; 65%]) were considered as treatment failures after three to six months. Individual modification of BSS from baseline is shown in Fig 2. At one month, 10 patients were evaluable for remission of diarrhoea. Among them, six patients (60%) had remission of diarrhoea at one month. Five of them maintained remission of diarrhoea over three to six months. One of the six responders had to stop somatostatin analogues because of hypoglycaemia and had a relapse of diarrhoea at the distant time point. Four patients had persistent diarrhoea at 1 month; among them, one stopped somatostatin analogue because of hypoglycaemia. Three patients who did not respond to somatostatin analogues at one month, continued and reached remission of diarrhoea at 3 to 6 months.

#### Number of stools per day

**Fig 3:**
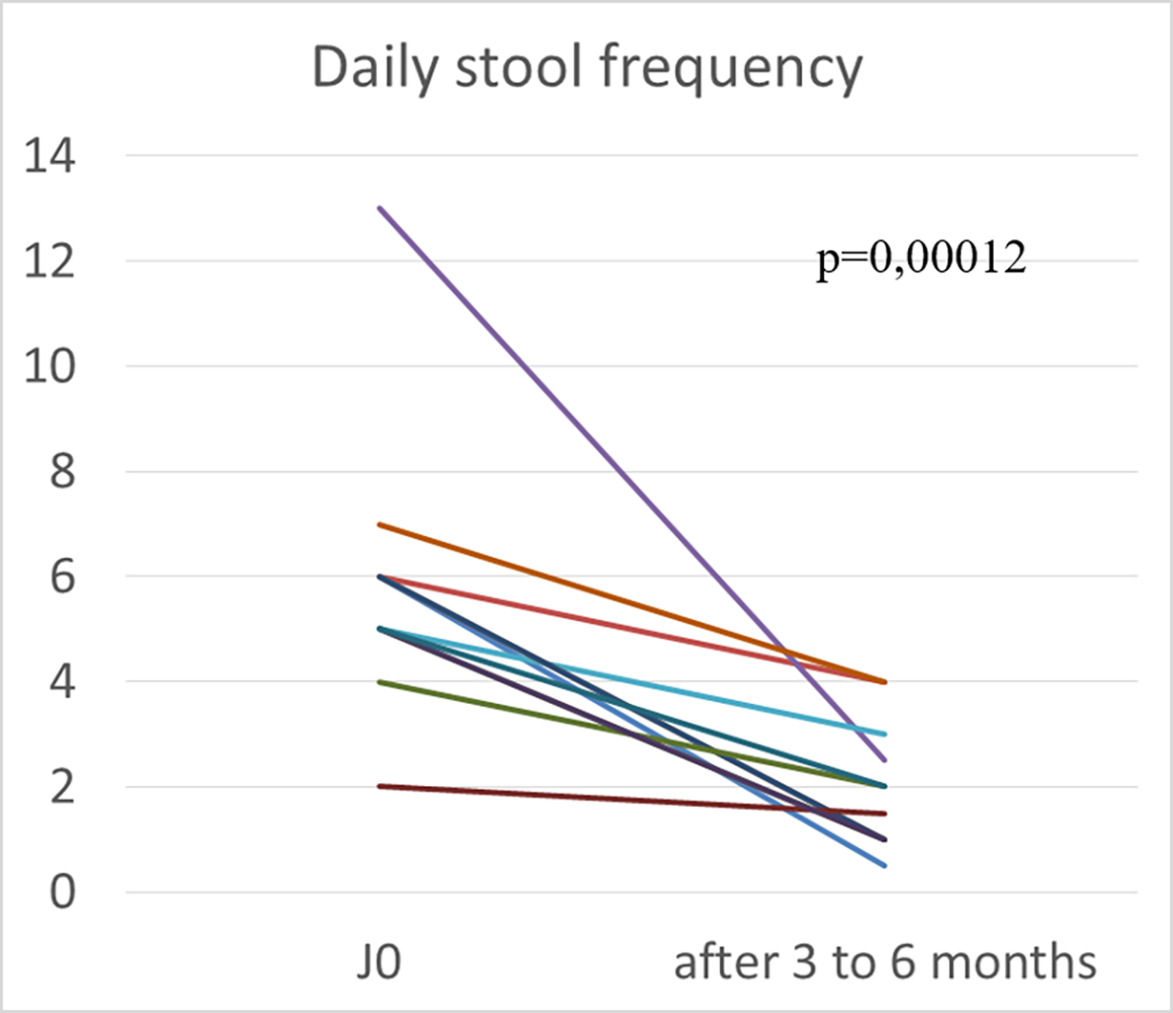
Evolution of number of stools per day, in individual patients with FAP and refractory diarrhoea, treated with somatostatin analogues. Y axis: number of stool per day. X axis: baseline, and after 3 to 6 months of treatment with somatostatin analogues. (n = 14).Median at baseline and after 3 to 6 months were 6 [2; 13] and 2 [0.5; 4], respectively. (p = 0.00012).

The number of stools per day decreased in all 14 patients. The median number of stools at baseline and after 3 to 6 months was 6 [2; 13] and 2 [0.5; 4], respectively (p = 0.00012; see Fig 3 for graphical representation).

#### Fecal incontinence

Twelve out of 14 patients (86%) had fecal incontinence at baseline, *vs* 8/14 (57%) after 3 to 6 months of treatment (p = 0.125).

Median pre-morbid body weight was 67.5 kg (47–120); it was 55 kg (34–76.5) at baseline and 56.3 kg (35–76), after 3 to 6 months of treatment (p = 0.12 *vs* baseline).

### Safety

Among the 20 patients who received somatostatin analogues as a treatment for diarrhoea, 16 were evaluable for safety. One patient underwent an orthotopic liver transplantation for liver failure secondary to a chronic liver transplant rejection, unrelated to somatostatin analogue. One patient died from cardiac arrest due to amyloid cardiomyopathy, unrelated to somatostatin analogue. Two patients had hypoglycemia, including one who was hospitalized for it. Both stopped somatostatin analogues. There was no case of symptomatic gallstone disease. One patient with a pacemaker, who had sustained improvement with somatostatin analogue, had an endocarditis due to *Staphylococcus epidermidis*, after 2 years of treatment. The injection site was presumed to be the source of infection. Nevertheless, due to clinical benefit on diarrhoea, somatostatin analogues were resumed.

## Discussion

In this study, somatostatin analogues led to remission of refractory diarrhoea in approximately two-third of patients with FAP (64%; [35%; 87%]), after 3 to 6 months of treatment. These drugs also reduced the median number of stools from 6 to 2, and tended to improve faecal continence.

Refractory diarrhoea is a disabling complication of FAP that seriously affects quality of life. This study suggests that somatostatin analogues improve stool frequency and consistence in patients with FAP and refractory diarrhoea. Somatostatin analogues could have palliated defective somatostatin signalling, due to somatostatin cell-depletion within the intestinal mucosa of patients. Furthermore, somatostatin analogues slow intestinal transit, reduce gastric, biliary and pancreatic secretion [27].

Serious adverse events attributable to somatostatin analogues were seen in three out of 14 patients (21%). Two patients had hypoglycaemia, and one had an endocarditis which was related to injection-site infection with a *Staphylococcus epidermidis*. Somatostatin analogues are known to trigger hyperglycaemia [28], and hypoglycaemia [24]. It is possible that our patients’ impaired nutritional status had contributed to the hypoglycaemia.

Our study has limitations inherent to its retrospective design. There was no control group. We assume that a spontaneous improvement would occur in no more than 20% of the patients. The observed remission rate of 64% (IC95% [0.31; 0.86]), after 3 to 6 months of somatostatin analogue would be higher than a theoretical proportion of 20% (binomial exact unilateral p<0.001). The superiority of octreotide would still be significant as compared with a theoretical spontaneous improvement of 39% (p = 0.05), which seems unlikely in these patients with a neurodegenerative disease who had failed symptomatic treatment. A prospective study would be more suitable to provide evidence for somatostatin analogue efficacy and safety in this setting. Yet, our study, as the first of its kind, suggests that a drug class, somatostatin analogues, may relief symptoms of a rare and devastating disease, refractory diarrhoea associated with FAP. The results provided allow to calculate an adequate number of patients to include in a prospective randomized trial.

## Conclusion

This study suggests that somatostatin analogues may benefit to patients with FAP and refractory diarrhoea. There is a relatively high frequency of adverse events, mostly glycaemic disorders. Our data should be confirmed by a prospective randomized trial.

## Supporting information

S1 Dataset (ZIP)
